# Utilisation and safety of catheter ablation of atrial fibrillation in public and private sector hospitals

**DOI:** 10.1186/s12913-021-06874-7

**Published:** 2021-08-28

**Authors:** Linh Ngo, Anna Ali, Anand Ganesan, Richard J Woodman, Robert Adams, Isuru Ranasinghe

**Affiliations:** 1 School of Clinical Medicine, The University of Queensland, Northside Clinical Unit, The Prince Charles Hospital, 627 Rode Road, Queensland 4032 Chermside, Australia; 2 Department of Cardiology, The Prince Charles Hospital, Chermside, Queensland Australia; 3 Cardiovascular Centre, E Hospital, Hanoi, Vietnam; 4 Discipline of Medicine, The University of Adelaide, South Australia Adelaide, Australia; 5 Department of Cardiovascular Medicine, Flinders Medical Centre, South Australia Bedford Park, Australia; 6 College of Medicine and Public Health, Flinders University, South Australia Adelaide, Australia; 7 Flinders Centre for Epidemiology and Biostatistics, College of Medicine and Public Health, Flinders University, South Australia Adelaide, Australia; 8 Respiratory and Sleep Services, Southern Adelaide Local Health Network, South Australia Adelaide, Australia

**Keywords:** Utilisation, Safety, Catheter ablation, Atrial fibrillation

## Abstract

**Background:**

Little is known about the utilisation and safety of catheter ablation of atrial fibrillation (AF) among public and private sector hospitals.

**Aims:**

To examine the uptake of AF ablations and compare procedural safety between the sectors.

**Method:**

Hospitalisation data from all public and private hospitals in four large Australian states (NSW, QLD, VIC and WA) were used to identify patients undergoing AF ablation from 2012 to 17. The primary endpoint was any procedure-related complications up to 30-days post-discharge. Logistic regression was used to evaluate the association between treatment at a public hospital and risk of complications adjusting for covariates.

**Results:**

Private hospitals performed most of the 21,654 AF ablations identified (*n* = 16,992, 78.5 %), on patients who were older (63.5 vs. 59.9y) but had lower rates of heart failure (7.9 % vs. 10.4 %), diabetes (10.2 % vs. 14.1 %), and chronic kidney diseases (2.4 % vs. 5.2 %) (all *p* < 0.001) than those treated in public hospitals. When compared with private hospitals, public hospitals had a higher crude rate of complications (7.25 % vs. 4.70 %, *p* < 0.001). This difference remained significant after adjustment (OR 1.74 [95 % CI 1.54–2.04]) and it occurred with both in-hospital (OR 1.83 [1.57–2.14]) and post-discharge (OR 1.39 [1.06–1.83]) complications, with certain complications including acute kidney injury (OR 5.31 [3.02–9.36]), cardiac surgery (OR 5.18 [2.19–12.27]), and pericardial effusion (OR 2.18 [1.50–3.16]).

**Conclusions:**

Private hospitals performed most of AF ablations in Australia with a lower rate of complications when compared with public hospitals. Further investigations are needed to identify the precise mechanisms of this observed difference.

**Supplementary Information:**

The online version contains supplementary material available at 10.1186/s12913-021-06874-7.

## Introduction

Atrial fibrillation (AF) affects millions of people worldwide and is associated with an increased risk of mortality, morbidity and significant economic burden [1]. Catheter ablation is a guidelines-recommended therapy to treat this debilitating condition [2] and in Australia, it is one of the fastest growing cardiovascular procedures whose annual number increased by 30.8 % per year [3]. Nevertheless, concerns still exist about procedural safety due to its associated risk of serious complications such as stroke, pericardial effusion or major bleeding [4]. Understanding these risks is critical to assist patients and physicians in their discussion regarding AF ablations.

Australia has a hybrid healthcare system in which public and private sectors coexist but little is known about the sector-wide differences in care outcomes [5]. A few studies have compared sector-wide performances of other services such as cardiac device implantation [6], cardiac surgery [7] and prelabour caesarean [8] and found considerable differences between sectors [7, 8], raising concerns about potential disparities with AF ablations. Given the rapid dissemination of this procedure, it is imperative to investigate the uptake of AF ablation and whether the safety is comparable in public and private health sectors. This information is important for patients and clinicians in their decision-making process and for hospitals and policy makers seeking to improve care quality.

Accordingly, we sought to characterise the patients undergoing catheter ablation of AF among public and private sector hospitals using hospitalisation data from several large states in Australia. We also examined the sector-wide differences in procedural complications to better understand the outcomes of this procedure in public and private sector hospitals.

## Methods

### Data source

We used the Admitted Patient Collection (APC) which records all inpatient and day-only admissions irrespective of age or funder. A standard set of variables is collected for each admission including patient demographics, primary and up to 50 secondary diagnoses coded per International Classification of Diseases, 10th revision Australian Modification (ICD-10-AM), up to 50 procedures coded per the Australian Classification of Health Interventions (ACHI), and the patient status at discharge. The data linkage units of each state established the linkages within the APC dataset and between the APC and Registry of Deaths, allowing us to identify hospital re-admissions to any hospital and post-procedural deaths including those occurring in community. The accuracy of linking health records using probabilistic matching techniques based on multiple patient identifiers has been reported to be greater than 99 % [9]. Coding of diagnoses and procedures in Australia has been validated to be reasonably accurate (> 85 %), especially for cardiovascular diagnoses and procedures [10]. We used data from New South Wales (NSW), Victoria (VIC), Queensland (QLD), and Western Australia (WA) as private hospital data for research are only available in these states.

### Study cohort

We included patients aged ≥ 18 years hospitalised with AF as the primary diagnosis and a procedure code of catheter ablation from 2012 to 2017 (refer to Supplemental Table S1 for full description of catheter ablation procedure and AF diagnosis codes). Such an approach to identify AF ablation using coded data has been shown to have 100 % specificity and 87.3 % sensitivity [11].

We excluded patients who had (1) secondary diagnosis of other arrhythmia; (2) current procedure code for a cardiac implantable electronic device (CIED) implantation or a diagnosis code for the presence of a cardiac device; (3) procedure code for open ablation; (4) patients who were discharged against medical advice; (5) had prior catheter ablation within 30 days to ensure complications were due to the index procedure, and (6) lacked 30-day post-discharge follow-up data (patients who underwent ablation after the 1st of December 2017).

### Outcomes

Our primary endpoint was the occurrence of any complication during the hospital stay or post-discharge (up to 30-days). *Procedure-related complications* included (i) death; (ii) cardiopulmonary failure and shock; (iii) stroke or transient ischemic attack (TIA); (iv) pericardial effusion; (v) haemothorax or pneumothorax; (vi) bleeding (*haemorrhage or hematoma formation, bleeding from major organs, or requirement for blood transfusion*); (vii) vascular injury or intervention; (viii) infections (*pneumonia, sepsis, or endocarditis*); (ix) pericarditis; (x) acute myocardial infarction; (xi) venous thromboembolism; (xii) acute kidney injury; (xiii) complications requiring cardiac surgery; and (xiv) complete atrioventricular (AV) block. *In-hospital complications* were identified based on the secondary diagnoses and procedure codes of the index hospitalisations. *Post-discharge complications* consisted of deaths or any hospital readmission with a complication coded as the primary diagnosis. Full description of complications and relevant codes are provided in Supplemental Table S2.

### Statistical Analysis

We presented discrete variables as frequencies and percentages, continuous variables as mean ± standard deviation if normally distributed, or as median and interquartile range otherwise. Differences between continuous variables were tested using student T-test or Mann-Whitney U test, while χ^2^ or Fisher’s exact test was used for discrete variables. Multiple events occurred in the same patient were counted once.

To compare procedural safety between two sectors, we used logistic regression to adjust for differences in patient characteristics. Variables considered for adjustment included age, gender, year of ablation, history of AF ablation in the preceding year, ablation of both atria, and a wide range of comorbidities. We identified patient comorbidities by using the Condition Category (CC) classification which groups ICD-10 codes into approximately 180 clinically meaningful conditions using diagnosis codes from the index admission and prior admissions within the preceding 12 months [12]. These candidate variables were backward eliminated until only those significantly associated with risk of procedural complications (*p* < 0.05) remained in the model.

#### Sensitivity analysis

We repeated our analysis with propensity score matching which is considered the optimal post-hoc method to minimise selection bias resulting from non-randomised allocation of measured covariates in an observational study [13]. The propensity score is the probability of being treated at a public hospital, estimated using a logistic regression model with patient age, gender, history of catheter ablation, ablation both atria, total length of stay, year of ablation and 180 comorbidities as independent covariates. Each patient treated in a public hospital was matched with another treated in private sector with similar propensity score without replacement using a caliper width of 0.01. The similarity of the matched groups was evaluated by calculating the standardised bias for each covariate [13], which reflects the difference in means (or medians) of a continuous variable or proportions of a categorical variable in two matched groups. A value < 5 % is generally considered acceptable [14]. Logistic regression was performed on the matched cohort with being treated in public hospitals as the only independent variable.

We also evaluated the strength that any confounding factor would need to nullify any observed difference between sectors by estimating the E value, which represents the association a confounder would need to have with both the intervention (treatment in a public hospital) and outcome (experiencing a procedural complication) to shift the lower limit of the estimated odd ratio (OR) across 1.0 [15].

Results were reported as OR and 95 % confidence intervals (CI) with private hospitals as the reference group. A two-tailed p value of < 0.05 was considered statistically significant. All analyses were performed using Stata version 16.0.

### Ethics approval and consent to participate

The Human Research Ethics Committees of all states granted ethical approval for the study including a waiver of informed consent for use of de-identified patient data. The study was approved by the University of Queensland and all methods were carried out in accordance with relevant human research ethics guidelines and local governance protocols.

## Results

### Study cohort selection

**Fig. 1:**
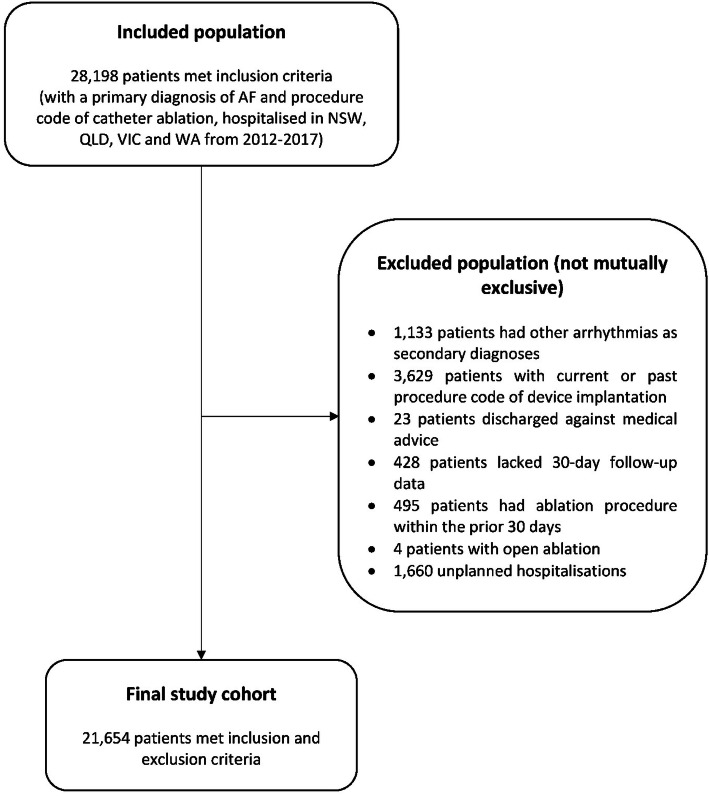
Study cohort selection. Abbreviation: AF = Atrial fibrillation. NSW = New South Wales, QLD = Queensland, VIC = Victoria, WA = Western Australia

We identified 28,198 patients meeting inclusion criteria (Fig. 1). The main reasons for exclusion were (not mutually exclusive): having current or past device implantation (3,629 patients) or being admitted as an acute hospitalisation (1,660 patients). The final study cohort consisted of 21,654 patients.

### Baseline characteristics of study cohort

The baseline characteristics of patients undergoing AF ablation are summarised in Table 1. The mean age (± SD) of the study cohort was 62.8 (± 11.2) years old with 46.8 % aged 65 or older. Females accounted for 30.3 % of patients. The median length of stay (LOS) for an AF ablation was one day (IQR: 1.0–2.0 days). Comorbidities were infrequent with hypertension (11.0 %) and diabetes (11.1 %) being the most common cardiac and non-cardiac comorbidities respectively.

**Table 1:** Baseline characteristics of patients undergoing AF ablation stratified by hospital sector

Variables	Overall cohort (*N* = 21,654)			Matched cohort (*N* = 8,868)		
	**Public hospitals (*N* = 4,662)n (%)**	**Private hospitals (*N* = 16,992)n (%)**	***P*value**	**Public hospitals (*N* = 4,434)n (%)**	**Private hospitals (*N* = 4,434)n (%)**	**Standardized bias**^a^**(%)**
**Patients’ demographics**						
Age (mean ± SD)	59.9 ± 12.4	63.5 ± 10.8	< 0.001	60.5 ± 11.9	60.3 ± 12.1	1.5
Age group, n (%)						
18–34	182 (3.9)	206 (1.2)	< 0.001	118 (2.7)	131 (3.0)	0.6
35–49	723 (15.5)	1,482 (8.7)		676 (15.3)	660 (14.9)	
50–64	2,002 (42.9)	6,915 (40.7)		1,927 (43.5)	1,907 (43.0)	
65–79	1,574 (33.8)	7,472 (44.0)		1,538 (34.7)	1,552 (35.0)	
≥ 80	180 (3.9)	917 (5.4)		175 (4.0)	184 (4.2)	
Female, n (%)	1,416 (21.6)	3,246 (21.5)	0.914	1,337 (30.2)	1,350 (30.5)	0.6
Median length of stay (IQR)	1.0 (1.0–1.0)	1.0 (1.0–1.0)	> 0.05	1.0 (1.0–1.0)	1.0 (1.0–2.0)	3.5
**Cardiovascular history**						
Hypertension	527 (11.3)	1,852 (10.9)	0.434	480 (10.8)	489 (11.0)	0.6
Heart failure	484 (10.4)	1,335 (7.9)	< 0.001	411 (9.3)	430 (9.7)	1.5
Valvular and rheumatic heart disease	122 (2.6)	659 (3.9)	< 0.001	110 (2.5)	107 (2.4)	0.4
Coronary artery disease	406 (8.7)	1,768 (10.4)	0.001	372 (8.4)	380 (8.6)	0.6
Vascular disease	84 (1.8)	261 (1.5)	0.199	65 (1.5)	65 (1.5)	0.0
Prior AF hospitalizations	2,838 (60.9)	10,782 (63.5)	0.001	2,664 (60.1)	2,623 (59.2)	1.9
Prior AF ablation	455 (9.8)	2,284 (13.4)	< 0.001	437 (9.9)	422 (9.5)	1.1
Prior stroke/TIA	56 (1.2)	226 (1.3)	0.492	56 (1.3)	51 (1.2)	1.0
**Non-cardiovascular comorbidities**						
Diabetes mellitus	655 (14.1)	1,738 (10.2)	< 0.001	604 (13.6)	586 (13.2)	1.2
Chronic lung diseases	108 (2.3)	244 (1.4)	< 0.001	78 (1.8)	79 (1.8)	0.2
Chronic kidney disease	240 (5.2)	412 (2.4)	< 0.001	182 (4.1)	187 (4.2)	0.6
History of pneumonia	126 (2.7)	299 (1.8)	< 0.001	102 (2.3)	104 (2.4)	0.3
Major cancer	40 (0.9)	105 (0.6)	0.075	32 (0.7)	36 (0.8)	1.1
End-stage liver disease	6 (0.1)	19 (0.1)	0.764	5 (0.1)	4 (0.1)	0.7
Haematological disorders	340 (5.9)	760 (3.6)	< 0.001	227 (5.1)	233 (5.3)	0.6
Dementia or senility	11 (0.2)	21 (0.1)	0.077	8 (0.2)	9 (0.2)	0.5
Drug or alcohol abuse, psychosis or dependence	154 (3.3)	161 (1.0)	< 0.001	97 (2.2)	95 (2.1)	10.3
Psychiatric disorders	83 (1.8)	202 (1.2)	0.002	57 (1.3)	49 (1.1)	1.5
Neurological disorders and paralysis	54 (1.2)	182 (1.1)	0.611	50 (1.1)	50 (1.1)	0.0
History of head injury	30 (0.6)	85 (0.5)	0.233	28 (0.6)	29 (0.7)	0.3
History of bone fracture	18 (0.4)	72 (0.4)	0.724	17 (0.4)	22 (0.5)	1.8
Skin ulcers	18 (0.4)	29 (0.2)	0.005	11 (0.3)	12 (0.3)	0.4
Urinary tract disorders and incontinence	217 (4.7)	698 (4.1)	0.100	193 (4.4)	201 (4.5)	0.9

Footnote: *SD* standardised deviation, *IQR* interquartile range, *AF* atrial fibrillation, *TIA* transient ischaemic attack

^a^Standardised bias (%) is the difference in the means (medians) of a continuous variable or the proportions of a categorical variable in the matched groups of patients treated in public and private hospitals

The private sector hospitals performed more than three-quarters (78.5 %) of all AF Ablations. Compared with patients treated at private hospitals, those that underwent AF ablation at public hospitals were significantly younger (mean age 59.9 vs. 63.5 years, *p* < 0.001) but had higher rate of comorbidities including heart failure (10.4 % vs. 7.9 %, *p* < 0.001), diabetes mellitus (14.1 % vs. 10.2 %, *p* < 0.001), chronic lung diseases (2.3 % vs. 1.4 %, p < 0.001), chronic kidney disease (5.2 % vs. 2.4 %, p < 0.001), pneumonia (2.7 % vs. 1.8 %, *p* < 0.001), and haematological disorders (5.9 % vs. 3.6 %, *p* < 0.001). Conversely, patients treated in private hospitals had higher rate of valvular heart disease (3.9 % vs. 2.6 %, *p* < 0.001), coronary artery disease (10.4 % vs. 8.7 %, *p* = 0.001), AF hospitalisations and catheter ablation in the preceding year (63.5 % vs. 60.9 %, *p* = 0.001 and 13.4 % vs. 9.8 %, p < 0.001 respectively).

### Association of hospital type and risk of procedural complications

The crude 30-day complication rate was higher in public hospitals compared with private hospitals (7.25 % vs. 4.70 %, *p* < 0.001) (Table 2). Cardiopulmonary failure (0.41 % vs. 0.12 %, *p* < 0.001), pericardial effusion (1.05 % vs. 0.52 %, *p* < 0.001), bleeding (3.99 % vs. 2.90 %, *p* < 0.001), pericarditis (0.54 % vs. 0.23 %, *p* = 0.001), acute kidney injury (0.64 % vs. 0.14 %, *p* < 0.001), and complications requiring cardiac surgery (0.24 % vs. 0.07 %, *p* = 0.002) also occurred more frequently in public facilities. The complication rates were significantly higher in public hospitals than private hospitals with regard to in-hospital (5.86 % vs. 3.63 %, *p* < 0.001) but not post-discharge (1.63 % vs. 1.29 %, *p* = 0.081) complications (refer to Supplemental Table S3 for rates of specific in-hospital and post-discharge complications in each sector).

**Table 2:** Major complications after catheter ablation for atrial fibrillation by hospital sector

Procedural complications	Overall cohort			Matched cohort		
	Public hospitals	Private hospitals	P value^*^	Public hospitals	Private hospitals	P value^*^
Any complications	338 (7.25)	798 (4.70)	*< 0.001*	328 (7.40)	180 (4.06)	*< 0.001*
In-hospital complications	273 (5.86)	616 (3.63)	*< 0.001*	265 (5.98)	141 (3.18)	*< 0.001*
Post-discharge complications	76 (1.63)	220 (1.29)	*0.081*	73 (1.65)	47 (1.06)	*0.017*
Death	4 (0.09)	10 (0.06)	*0.518*	4 (0.09)	4 (0.09)	*1.000*
Cardiopulmonary failure and shock	19 (0.41)	20 (0.12)	*< 0.001*	18 (0.41)	7 (0.16)	*0.028*
Stroke/TIA	10 (0.21)	38 (0.22)	*0.906*	10 (0.23)	5 (0.11)	*0.196*
Pericardial effusion	49 (1.05)	89 (0.52)	*< 0.001*	46 (1.04)	18 (0.41)	*< 0.001*
*Pericardiocentesis*	27 (0.58)	57 (0.34)	*0.018*	25 (0.56)	13 (0.29)	*0.051*
Hemothorax/pneumothorax	6 (0.13)	23 (0.14)	*0.912*	6 (0.14)	5 (0.11)	*0.763*
Bleeding	186 (3.99)	492 (2.90)	*< 0.001*	181 (4.08)	113 (2.55)	*< 0.001*
*Postprocedural hemorrhage or hematoma*	143 (3.07)	378 (2.22)	*0.001*	140 (3.16)	83 (1.87)	*< 0.001*
*Bleeding from other sites*	32 (0.69)	86 (0.51)	*0.139*	32 (0.72)	22 (0.50)	*0.172*
*Bleeding requiring blood transfusion*	28 (0.60)	69 (0.41)	*0.078*	26 (0.59)	18 (0.41)	*0.227*
Vascular injury	14 (0.30)	32 (0.19)	*0.141*	14 (0.32)	7 (0.16)	*0.126*
Post-procedural infection	27 (0.58)	65 (0.38)	*0.067*	24 (0.54)	15 (0.34)	*0.149*
Pericarditis	25 (0.54)	39 (0.23)	*0.001*	25 (0.56)	10 (0.23)	*0.011*
Procedure-related AMI	5 (0.11)	17 (0.10)	*0.800*	5 (0.11)	3 (0.07)	*0.726*
Venous thromboembolism	3 (0.06)	13 (0.08)	*1.000*	3 (0.07)	1 (0.02)	*0.625*
Acute kidney injury	30 (0.64)	24 (0.14)	*< 0.001*	27 (0.61)	5 (0.11)	*< 0.001*
Complications requiring cardiac surgery	11 (0.24)	12 (0.07)	*0.002*	10 (0.23)	2 (0.05)	*0.021*
Complete AV block	11 (0.24)	39 (0.23)	*0.935*	11 (0.25)	13 (0.29)	*0.683*

Footnote: ^*^p value from chi square or Fisher’s exact test comparison

**Fig. 2:**
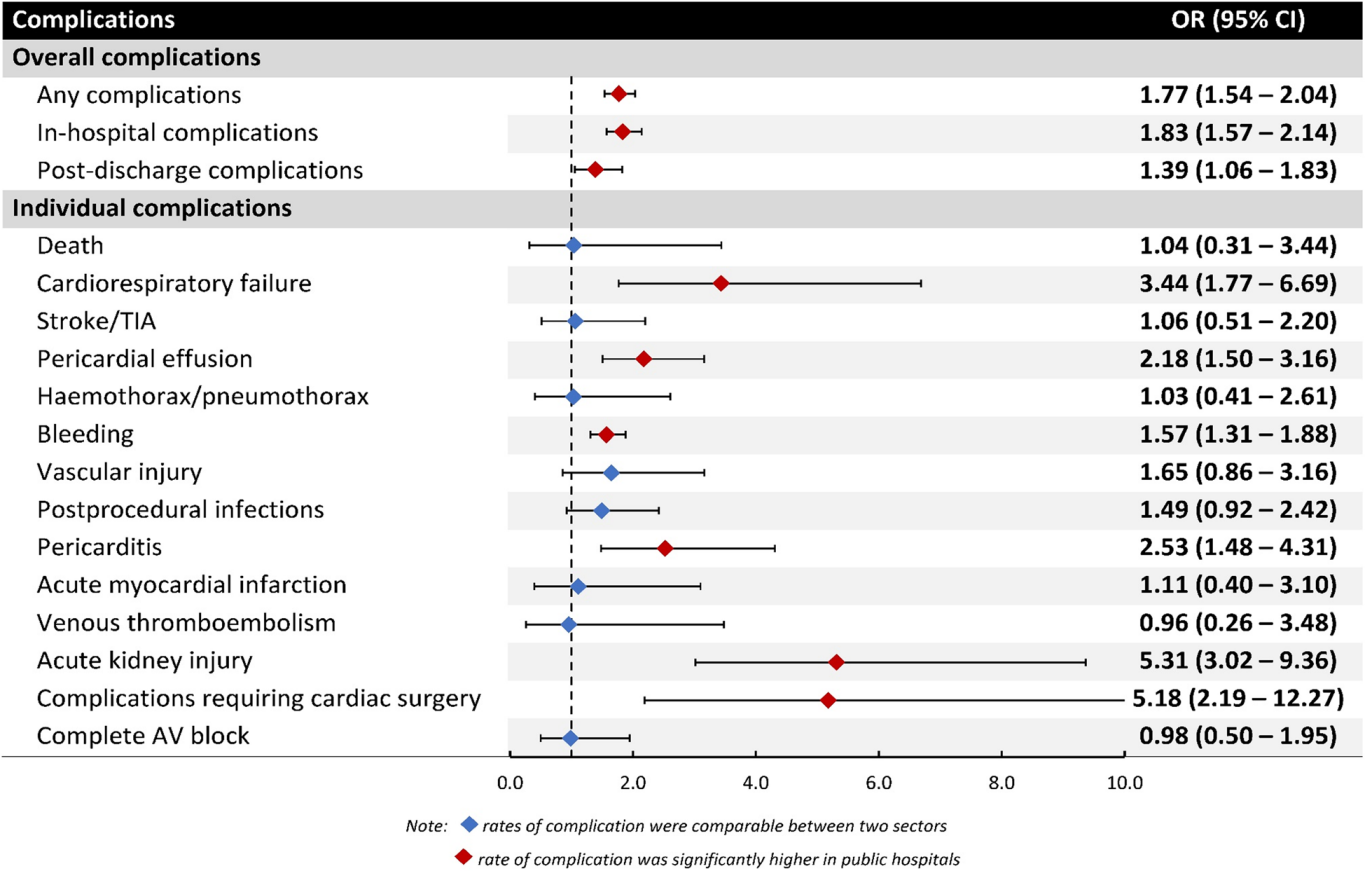
Adjusted risk of procedural complications based on hospital section (private hospitals as the reference) after logistic regression. Abbreviation: CI = Confidence Intervals; OR = Odd Ratio

After adjusting for differences in patient characteristics, ablation at a public hospital was associated with a higher risk of complications compared with treatment at a private hospital (OR 1.77, 95 % CI 1.54–2.04,  < 0.001) (Fig. 2). When individual complications were considered, this increase was mainly driven by higher odds of acute kidney injury (OR 5.31, 95 % CI 3.02–9.36, *p* < 0.001), complications requiring cardiac surgery (OR 5.18, 95 % CI 2.19–12.27, *p* < 0.001), cardiorespiratory failure (OR 3.44, 95 % CI 1.77–6.69, *p* < 0.001), pericarditis (OR 2.53, 95 % CI 1.48–4.31, *p* = 0.001), pericardial effusion (OR 2.18, 95 % CI 1.50–3.16, *p* < 0.001), and bleeding (OR 1.57, 95 % CI 1.31–1.88, *p* < 0.001) (Fig. 2). The higher rates of complications among public hospitals occurred with both in-hospital (OR 1.83, 95 %CI 1.57–2.14, *p* < 0.001) and post-discharge (OR 1.39, 95 % CI 1.06–1.83, *p* = 0.019) complications (refer to supplemental tables S4 and S5 for more details).

### Sensitivity analysis

**Fig. 3:**
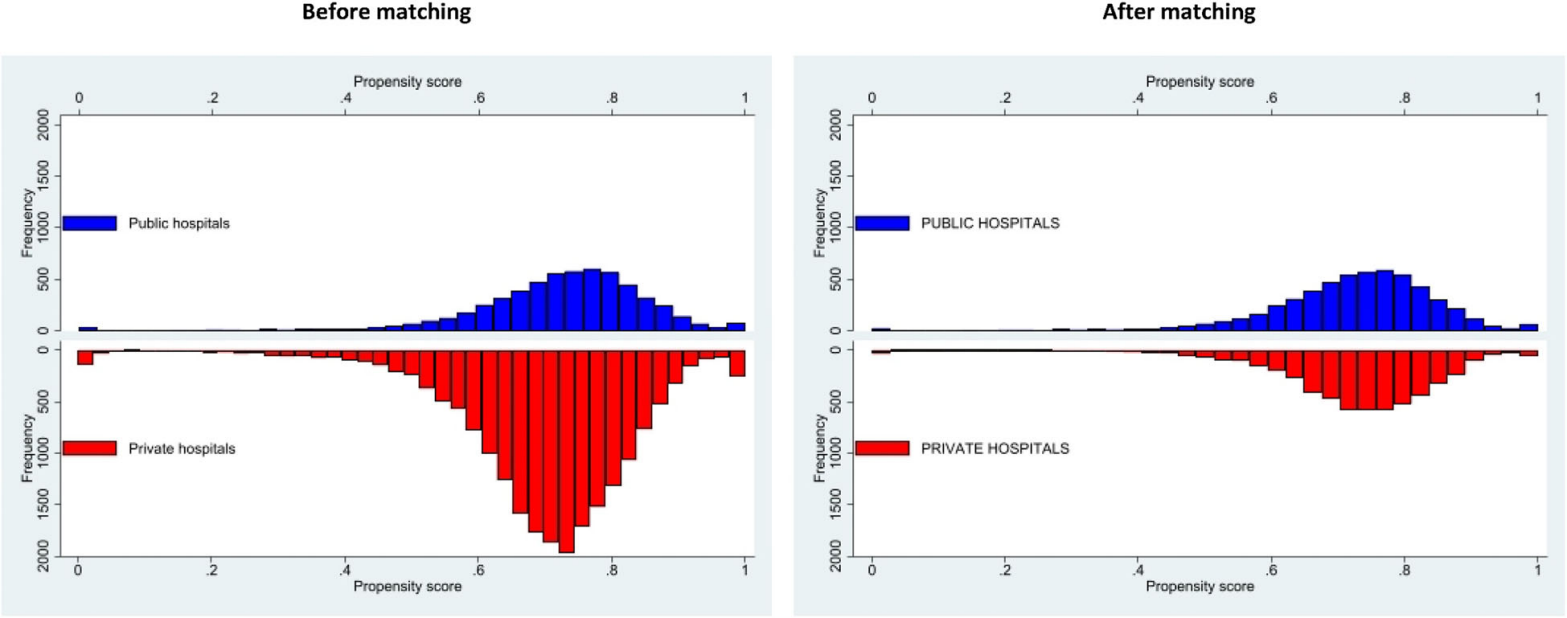
Distribution of propensity score in public and private hospitals before and after matching. The figure shows that after matching, the distributions of propensity score were balanced between public and private hospitals, suggesting similar baseline characteristics in the matched groups

**Fig. 4:**
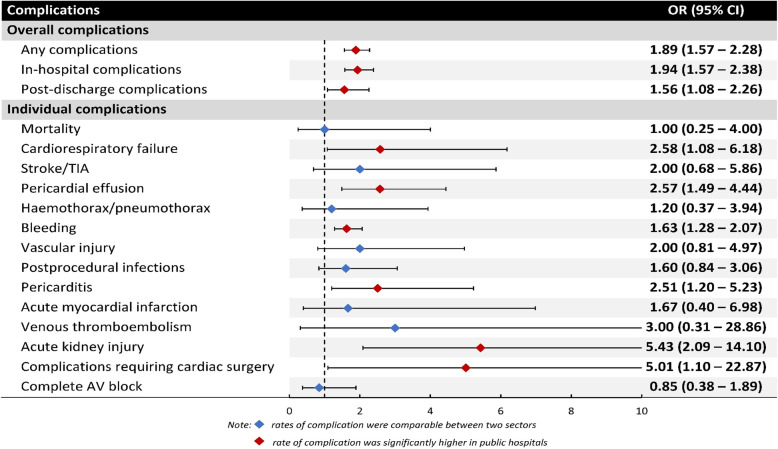
Adjusted risk of procedural complications based on hospital sector (private hospitals are the reference) after propensity score matching. Abbreviation: CI = Confidence Intervals; OR = Odd Ratio

The matched cohort consisted of two groups of 4,434 patients each with closely matched patient characteristics as indicated by similarity in the distribution of the propensity score after matching (Fig. 3) as well as a median standardised bias of 0.8 % (IQR 0.3 − 1.3 %). Consistent with logistic regression, in the matched cohort, patients treated at public hospitals also experienced higher overall rate of complications (OR 1.95, 95 % CI 1.61–2.35) including in-hospital (OR 1.94, 95 % CI 1.57–2.38) and post-discharge (OR 1.56, 95 % CI 1.08–2.26) complications (Table 2; Fig. 4). When individual complications were considered, public hospitals also had higher rate of cardiopulmonary failure and shock (OR 2.58, 95 % CI 1.08–6.18), pericardial effusion (OR 2.57, 95 % CI 1.49–4.44), bleeding (OR 1.63, 95 % CI 1.28–2.07), pericarditis (OR 2.51, 95 % CI 1.20–5.23), acute kidney injury (OR 5.43, 95 % CI 2.09–14.10), and complications requiring cardiac surgery (OR 5.01, 95 % CI 1.10–22.87).

The estimated E value to shift the lower limit (1.54) of the estimated OR across 1.0 was 2.45, meaning that a confounder would need to be 2.45 times more common in public hospitals *and* be associated with a 2.45-time higher risk of complications, which is considered unlikely [15].

## Discussion

In this population-based study, we found that more than three-quarters of AF ablations in Australia were performed in private sector hospitals and there were significant differences between sectors in procedural safety. Specifically, patients undergoing AF ablation at public hospitals experienced higher risk of complications which occurred with certain complications. These findings suggest a possible disparity in procedural safety between the two sectors, although these differences might also be explained by unmeasured confounders such as greater complexity of AF ablations performed at public hospitals.

Although nearly half of hospitals in Australia are private facilities [16], little is known about differences in outcomes between public and private sector hospitals. Our study represents the first evaluation of the sector-wide differences in the uptake and safety of AF ablations. Unlike other cardiovascular interventions where both sectors have nearly equal utilisation such as cardiac device implantation (48.7 % performed in private hospitals vs. 51.3 % in public hospitals) [6] and elective coronary artery bypass grafting (CABG) (46.1 % vs. 53.9 %) [7], the majority of AF ablations was performed in private hospitals. Nevertheless, the pattern of patient selection is consistent throughout studies with private hospitals tending to treat patients with less comorbidities than their public counterparts [6, 7]. Patient outcomes, on the other hand, are less consistent. Public hospitals are reported to have higher rate of postoperative sepsis (2.94 % vs. 1.33 %, p < 0.001) and in-hospital mortality (0.99 % vs. 0.61 %) after CABG surgery compared with private facilities [7] but rates of complications following cardiac device implantation are comparable between sectors (OR 0.92, 95 % CI 1.04–1.00, p = 0.06) [6]. And while we found a higher risk of overall and several complications in public hospitals, rates of deaths and stroke were low and comparable between sectors. Collectively, these findings provide insights to the practice and performance of AF ablation among private and public sector hospitals in Australia.

Several explanations exist for the observed sector-wide differences in procedural safety of AF ablations. A systemic difference in coding practices between two sectors could lead to disparity in outcomes. However, private hospitals usually have more financial incentive than public facilities to code complications appropriately as they entirely depend on reimbursement. Moreover, prior studies of cardiac device complications showed comparable complication rates between sectors [6], making systematic differences in coding unlikely. Given that measured covariates including patient comorbidities were adjusted for, with both logistic regression and propensity score matching, the observed disparity may suggest sector-wide disparities in the care process including procedural techniques, anticoagulation strategy, or post-discharge care. Indeed, the differences were seen for complications that are preventable by optimising procedural technique such as pericardial effusion, bleeding, and acute kidney injury. Unmeasured confounders such as procedural complexity and operator experience may also contribute. As public hospitals treated higher risk patients, they may perform more complex procedures compared with private hospitals. And while this procedure might be solely performed by senior operators in private sector, some ablations in public sector may be carried out by less-experienced trainees who are reported to have higher complication rate compared with their senior colleagues (who performed > 25 ablations per year) [17]. Further studies, preferably well-designed multicentre registries, are needed to elucidate the causes of these sector-wide differences.

Based on our findings, patients could be better-informed about the sector-wide differences in risk of procedural complications when considering AF ablations. Both public and private hospitals could also use these results to establish a targeted strategy to improve care quality. Specifically, public hospitals should focus on reducing complications that were driving the disparity like pericardial effusion, bleeding, and acute kidney injury. Potential measures to reduce these complications include using ultrasound to guide vascular access [18], adequate hydration with intravenous fluid to reduce contrast-induced acute kidney injury [19], or implementing safety checklists to reduce procedural complications [20]. Private hospitals, on the other hand, could further improve procedural safety by focusing on the most common complications like bleeding and pericardial effusion. Moreover, given that the private sector performed most of the AF ablations, greater reporting of procedural outcomes across both public and private sector hospitals is crucial to inform AF ablations practice in Australia.

Our study has several limitations that should be considered. This study used administrative data, which are generally considered less granular and accurate than data collected specifically for research purposes. Nevertheless, reasonable accuracy (> 85 %) has been reported for the coding of diseases and procedures compared with medical records in Australian setting [10]. Data were aggregated for private hospitals and unavailable for operator, so we were unable to examine hospital or proceduralist-specific performance. We were unable to adjust for potential confounders including medications, ablation energy (radiofrequency vs. cryoablation), operator experience, of the procedural technique such as the use of vascular ultrasound or intracardiac echocardiography, procedural time, or ablation lesions. Nevertheless, our sensitivity analysis shows that a confounding factor is unlikely to explain away the observed sector-wide difference. Our study also could not capture some complications that do not have specific diagnosis codes including phrenic nerve injury, pulmonary vein stenosis or atrio-oesophageal fistula. These complications, however, are rare, usually present beyond 30 days post-discharge, and only a few cases of phrenic nerve injury and pulmonary vein stenosis require treatment [21, 22]. The incidence of atrio-oesophageal fistula is also exceedingly rare [23].

## Conclusions

Most catheter ablation procedures for AF in Australia are performed in private hospitals. Compared with private sector hospitals, patients undergoing AF ablation at public hospitals experience a higher risk of complications that occurred with certain types of complications. Whether these differences can be explained by hospital level characteristics, disparity in care quality or other factors requires further investigation.

## Supplementary Information

**Additional file 1:**

## Data Availability

The data that support the findings of this study are available from the respective Data Custodians of the states included in our analysis but restrictions apply to the availability of these data, which were used under license for the current study, and so are not publicly available. Data are however available upon ethics applications to the respective states.
