# Supplementary material for: Utilisation and safety of catheter ablation of atrial fibrillation in public and private sector hospitals
Source: BMC Health Serv Res. 2021 Aug 28;21:883. doi: 10.1186/s12913-021-06874-7 (PMC8400841; doi:10.1186/s12913-021-06874-7)
Supplement: Supplementary file 1 — Additional file 1: [file 12913_2021_6874_MOESM1_ESM.docx]

**SUPPLEMENTARY APPENDIX**

**Utilisation and safety of catheter ablation of atrial fibrillation** **in public and private sector hospitals**

**Supplemental Tables**

Table S1: ICD-10 AM and ACHI codes used to identify patients undergoing catheter ablation for atrial fibrillation

Table S2: Diagnoses and procedure codes used to identify in-hospital and post-discharge complications

Table S3: Rates of in-hospital and post-discharge complications stratified by hospital’s type

Table S4: Variables included in the logistic regression model to evaluate the association between ablation at a public hospital and risk of complications (treatment at a private hospital as the reference group).

Table S5: Association between treatment at a public hospital and the risk of experiencing in-hospital and post-discharge complications with logistic regression (treatment at a private hospital as the reference group).

**Table S1: ICD-10 AM and ACHI codes used to identify patients undergoing catheter ablation of atrial fibrillation**

| **GROUP** | **DISEASE/PROCEDURE** | **ICD10-AM/ACHI codes** |
| --- | --- | --- |
| Inclusion | Atrial fibrillation | I48, I48.0, I48.1, I48.2, I48.9 |
|  | Catheter ablation | 38287-01, 38287-02, 38290-01 |
| Exclusion | Atrial flutter | I48.3, I48.4 |
|  | Pre-excitation syndrome | I45.6 |
|  | Supra-ventricular tachycardia | I47.1 |
|  | Ventricular tachycardia | I47.2, I49.0 |
|  | Premature beats | I49.1, I49.2, I49.3, I49.4 |
|  | Other arrhythmias | I47, I47.0, I48, I49.8, I49.9, R00.0 |
|  | Presence of a cardiac device | Z95.0 |
|  | Pacemaker implantation | 38353-00 |
|  | Cardiac defibrillator implantation | 38393-00 |
|  | Open ablation | 38287-03, 38287-04, 38290-02 |

**Table S2: Diagnoses and procedure codes used to identify in-hospital and post-discharge complications**

| **COMPLICATIONS** | **ICD-10 AM or ACHI codes** | **Code description** |  |
| --- | --- | --- | --- |
| **Cardiopulmonary failure and shock** | |  |  |
| Cardiac arrest | I46 | Cardiac arrest |  |
|  | I46.0 | Cardiac arrest with successful resuscitation |  |
|  | I46.9 | Cardiac arrest, unspecified |  |
|  | I46.1 | Sudden cardiac death, so described |  |
| Acute respiratory failure | J96.0 | Acute respiratory failure |  |
|  | J96.00 | Acute respiratory failure, type I |  |
|  | J96.01 | Acute respiratory failure, type II |  |
|  | J96.09 | Acute respiratory failure type unspecified |  |
| Shock | R57.x | Cardiogenic shock |  |
|  | T81.1 | Shock during or resulting from a procedure, not elsewhere classified |  |
|  | T88.2 | Shock due to anaesthesia |  |
|  | T78.2 | Anaphylactic shock, unspecified |  |
|  | T80.5 | Anaphylactic shock due to serum |  |
|  | T88.6 | Anaphylactic shock due to adverse effect of correct drug or medicament properly administered |  |
| Cardiorespiratory resuscitation | 92052-00 | Cardiopulmonary resuscitation |  |
| **Stroke/Transient ischemic attack** |  |  |  |
| Stroke | I64 | Stroke, not specified as haemorrhage or infarction |  |
|  | I63 | Cerebral infarction |  |
|  | I63.0 | Cerebral infarction due to thrombosis of precerebral arteries |  |
|  | I63.1 | Cerebral infarction due to embolism of precerebral arteries |  |
|  | I63.2 | Cerebral infarction due to unspecified occlusion or stenosis of precerebral arteries |  |
|  | I63.3 | Cerebral infarction due to thrombosis of cerebral arteries |  |
|  | I63.4 | Cerebral infarction due to embolism of cerebral arteries |  |
|  | I63.5 | Cerebral infarction due to unspecified occlusion or stenosis of cerebral arteries |  |
|  | I63.6 | Cerebral infarction due to cerebral venous thrombosis, nonpyogenic |  |
|  | I63.8 | Other cerebral infarction |  |
|  | I63.9 | Cerebral infarction, unspecified |  |
|  | I61 | Intracerebral haemorrhage |  |
|  | I61.0 | Intracerebral haemorrhage in hemisphere, subcortical |  |
|  | I61.1 | Intracerebral haemorrhage in hemisphere, cortical |  |
|  | I61.2 | Intracerebral haemorrhage in hemisphere, unspecified |  |
|  | I61.3 | Intracerebral haemorrhage in brain stem |  |
|  | I61.4 | Intracerebral haemorrhage in cerebellum |  |
|  | I61.5 | Intracerebral haemorrhage, intraventricular |  |
|  | I61.6 | Intracerebral haemorrhage, multiple localised |  |
|  | I61.8 | Other intracerebral haemorrhage |  |
|  | I61.9 | Intracerebral haemorrhage, unspecified |  |
|  | I62 | Other nontraumatic intracranial haemorrhage |  |
|  | I62.0 | Subdural haemorrhage (acute)(nontraumatic) |  |
|  | I62.1 | Nontraumatic extradural haemorrhage |  |
|  | I62.9 | Intracranial haemorrhage (nontraumatic), unspecified |  |
|  | I60 | Subarachnoid haemorrhage |  |
|  | I60.0 | Subarachnoid haemorrhage from carotid siphon and bifurcation |  |
|  | I60.1 | Subarachnoid haemorrhage from middle cerebral artery |  |
|  | I60.2 | Subarachnoid haemorrhage from anterior communicating artery |  |
|  | I60.3 | Subarachnoid haemorrhage from posterior communicating artery |  |
|  | I60.4 | Subarachnoid haemorrhage from basilar artery |  |
|  | I60.5 | Subarachnoid haemorrhage from vertebral artery |  |
|  | I60.6 | Subarachnoid haemorrhage from other intracranial arteries |  |
|  | I60.7 | Subarachnoid haemorrhage from intracranial artery, unspecified |  |
|  | I60.8 | Other subarachnoid haemorrhage |  |
|  | I60.9 | Subarachnoid haemorrhage, unspecified |  |
| Transient ischemic attack | G45 | Transient cerebral ischaemic attacks and related syndromes |  |
|  | G45.0 | Vertebro-basilar artery syndrome |  |
|  | G45.1 | Carotid artery syndrome (hemispheric) |  |
|  | G45.2 | Multiple and bilateral precerebral artery syndromes |  |
|  | G45.3 | Amaurosis fugax |  |
|  | G45.4 | Transient global amnesia |  |
|  | G45.8 | Other transient cerebral ischaemic attacks and related syndromes |  |
|  | G45.9 | Transient cerebral ischaemic attack, unspecified |  |
| **Perforation injury** | |  |  |
| Pericardial effusion | I31.2 | Haemopericardium, not elsewhere classified |  |
|  | I31.3 | Pericardial effusion (noninflammatory) |  |
| Pericardiocentesis | 3835900 | Pericardiocentesis |  |
|  | 3845000 | Transthoracic drainage of pericardium |  |
|  | 3845001 | Thoracoscopic drainage of pericardium |  |
|  | 3845200 | Subxyphoid drainage of pericardium |  |
| Haemothorax or pneumothorax | J93.2 | Iatrogenic pneumothorax |  |
|  | J93 | Pneumothorax |  |
|  | J93.9 | Pneumothorax, unspecified |  |
|  | J93.8 | Other pneumothorax |  |
| Thoracentesis | 3880000 | Diagnostic thoracentesis |  |
|  | 3880300 | Therapeutic thoracentesis |  |
|  | 3880600 | Insertion of intercostal catheter for drainage |  |
| Atrio-oesophageal fistula | K22.3 | Perforation of oesophagus |  |
| Mediastinitis | J85 | Abscess of lung and mediastinum |  |
|  | J85.3 | Abscess of mediastinum |  |
| **Any bleeding** | |  |  |
| Post-procedural haemorrhage/hematoma | R58 | Haemorrhage, not elsewhere classified |  |
|  | T81.0 | Haemorrhage and haematoma complicating a procedure, not elsewhere classified |  |
|  | Y60.5 | Unintentional cut, puncture, perforation or haemorrhage during heart catheterisation |  |
| Bleeding from other sites (GI, pulmonary, urinary, unspecified) | K92.2 | Gastrointestinal haemorrhage, unspecified |  |
|  | I98.3 | Esophageal varices with bleeding |  |
|  | K22.6 | Gastro-oesophageal laceration-haemorrhage syndrome |  |
|  | K25.0, 25.2, 25.4, 25.6 | Gastric ulcer with haemorrhage |  |
|  | K26.0, 26.2, 26.4, 26.6 | Duodenal ulcer with haemorrhage |  |
|  | K27.0, 27.2, 27.4, 27.6 | Peptic ulcer with haemorrhage |  |
|  | K28.0, 28.4, 28.6 | Gastrojejunal ulcer with haemorrhage |  |
|  | K29.0 | Acute haemorrhagic gastritis |  |
|  | K62.5 | Haemorrhage of anus and rectum |  |
|  | K66.1 | Hemoperitoneum |  |
|  | K92.0 | Hematemesis |  |
|  | K92.1 | Melena |  |
|  | R04.0 | Epistaxis |  |
|  | R04.1 | Haemorrhage from throat |  |
|  | R04.2 | Haemoptysis |  |
|  | R04.8 | Haemorrhage from other sites in respiratory passages |  |
|  | R04.9 | Haemorrhage from respiratory passages, unspecified |  |
|  | N02.x | Recurrent and persistent haematuria |  |
|  | R31.0 | Unspecified haematuria |  |
|  | D62 | Acute post haemorrhagic anaemia |  |
| Bleeding requiring blood transfusion | Z51.3 | Blood transfusion without reported diagnosis |  |
|  | 13706-01 | Administration of whole blood |  |
|  | 13706-02 | Administration of packed cells |  |
| **Vascular injury** |  |  |  |
| Vascular injury | I72.4 | Aneurysm and dissection of artery of lower extremity |  |
|  | I77.0 | Arteriovenous fistula, acquired |  |
|  | T81.7 | Vascular complications following a procedure, not elsewhere classified |  |
| Surgical repair | 33142-00 | Repair of false aneurysm in femoral artery |  |
|  | 33139-00 | Repair of false aneurysm in iliac artery |  |
|  | 34121-00 | Repair of simple arteriovenous fistula of extremity with restoration of continuity |  |
|  | 34121-01 | Repair of complex arteriovenous fistula of extremity with restoration of continuity |  |
| Vascular intervention | 45027-01 | Administration of agent into vascular anomaly |  |
|  | 33116-00 | Endovascular repair of aneurysm |  |
| **Post-procedural infections** | |  |  |
| Sepsis | T81.42 | Sepsis following a procedure |  |
|  | U90 | Healthcare associated infections |  |
|  | U90.0 | Healthcare associated Staphylococcus aureus bacteraemia |  |
| Pneumonia | J13 | Pneumonia due to Streptococcus pneumoniae |  |
|  | J14 | Pneumonia due to Haemophilus influenzae |  |
|  | J15 | Bacterial pneumonia, not elsewhere classified |  |
|  | J15.0 | Pneumonia due to Klebsiella pneumoniae |  |
|  | J15.1 | Pneumonia due to Pseudomonas |  |
|  | J15.2 | Pneumonia due to staphylococcus |  |
|  | J15.3 | Pneumonia due to streptococcus, group B |  |
|  | J15.4 | Pneumonia due to other streptococci |  |
|  | J15.5 | Pneumonia due to Escherichia coli |  |
|  | J15.6 | Pneumonia due to other Gram-negative bacteria |  |
|  | J15.7 | Pneumonia due to Mycoplasma pneumoniae |  |
|  | J15.8 | Other bacterial pneumonia |  |
|  | J15.9 | Bacterial pneumonia, unspecified |  |
|  | J18 | Pneumonia, organism unspecified |  |
|  | J18.0 | Bronchopneumonia, unspecified |  |
|  | J18.1 | Lobar pneumonia, unspecified |  |
|  | J18.2 | Hypostatic pneumonia, unspecified |  |
|  | J18.8 | Other pneumonia, organism unspecified |  |
|  | J18.9 | Pneumonia, unspecified |  |
| Endocarditis | I33 | Acute and subacute endocarditis |  |
|  | I33.0 | Acute and subacute infective endocarditis |  |
|  | I33.9 | Acute endocarditis, unspecified |  |
|  | I38 | Endocarditis, valve unspecified |  |
| **Pericarditis** |  |  |  |
| Pericarditis | I30 | Acute pericarditis |  |
|  | I30.0 | Acute nonspecific idiopathic pericarditis |  |
|  | I30.1 | Infective pericarditis |  |
|  | I30.8 | Other forms of acute pericarditis |  |
|  | I30.9 | Acute pericarditis, unspecified |  |
|  | I24.1 | Dressler's syndrome |  |
| **Post-procedural acute myocardial infarction** | |  |  |
| Acute myocardial infarction | I21 | Acute myocardial infarction |  |
|  | I21.0 | Acute transmural myocardial infarction of anterior wall |  |
|  | I21.1 | Acute transmural myocardial infarction of inferior wall |  |
|  | I21.2 | Acute transmural myocardial infarction of other sites |  |
|  | I21.3 | Acute transmural myocardial infarction of unspecified site |  |
|  | I21.4 | Acute subendocardial myocardial infarction |  |
|  | I21.9 | Acute myocardial infarction, unspecified |  |
| **Venous thromboembolism** |  |  |  |
| Pulmonary embolism | I26 | Pulmonary embolism |  |
|  | I26.0 | Pulmonary embolism with mention of acute cor pulmonale |  |
|  | I26.8 | Iatrogenic pulmonary embolism |  |
|  | I26.9 | Pulmonary embolism without mention of acute cor pulmonale |  |
| Deep vein thrombosis | I82 | Other venous embolism and thrombosis |  |
|  | I82.2 | Embolism and thrombosis of vena cava |  |
|  | I82.3 | Embolism and thrombosis of renal vein |  |
|  | I82.8 | Embolism and thrombosis of other specified veins |  |
|  | I82.9 | Embolism and thrombosis of unspecified vein |  |
| **Post-procedural acute kidney injury** |  |  |  |
| Acute kidney injury | N99.0 | Postprocedural kidney failure |  |
|  | N17 | Acute kidney failure |  |
|  | N17.0 | Acute kidney failure with tubular necrosis |  |
|  | N17.1 | Acute kidney failure with acute cortical necrosis |  |
|  | N17.2 | Acute kidney failure with medullary necrosis |  |
|  | N17.8 | Other acute kidney failure |  |
|  | N17.9 | Acute kidney failure, unspecified |  |
| **Complete heart block** | |  |  |
| Complete heart block | I44.2 | Atrioventricular block, complete |  |
| **Complications requiring cardiac surgery** | |  |  |
| Coronary artery bypass graft | 38497-00 | Coronary artery bypass, using 1 saphenous vein graft |  |
|  | 38497-01 | Coronary artery bypass, using 2 saphenous vein grafts |  |
|  | 38497-02 | Coronary artery bypass, using 3 saphenous vein grafts |  |
|  | 38497-03 | Coronary artery bypass, using >= 4 saphenous vein grafts |  |
|  | 38497-04 | Coronary artery bypass, using 1 other venous graft |  |
|  | 38497-05 | Coronary artery bypass, using 2 other venous grafts |  |
|  | 38497-06 | Coronary artery bypass, using 3 other venous grafts |  |
|  | 38497-07 | Coronary artery bypass, using >= 4 other venous grafts |  |
|  | 38500-00 | Coronary artery bypass, using 1 LIMA graft |  |
|  | 38503-00 | Coronary artery bypass, using >= 2 LIMA grafts |  |
|  | 38500-01 | Coronary artery bypass, using 1 RIMA graft |  |
|  | 38503-01 | Coronary artery bypass, using >= 2 RIMA grafts |  |
|  | 38500-02 | Coronary artery bypass, using 1 radial artery graft |  |
|  | 38503-02 | Coronary artery bypass, using >= 2 radial artery grafts |  |
|  | 38500-03 | Coronary artery bypass, using 1 epigastric artery graft |  |
|  | 38503-03 | Coronary artery bypass, using >= 2 epigastric artery grafts |  |
|  | 38500-04 | Coronary artery bypass, using 1 other arterial graft |  |
|  | 38503-04 | Coronary artery bypass, using >= 2 other arterial grafts |  |
|  | 38500-05 | Coronary artery bypass, using 1 composite graft |  |
|  | 38503-05 | Coronary artery bypass, using >= 2 composite grafts |  |
|  | 90201-00 | Coronary artery bypass, using 1 other graft, not elsewhere classified |  |
|  | 90201-01 | Coronary artery bypass, using 2 other grafts, not elsewhere classified |  |
|  | 90201-02 | Coronary artery bypass, using 3 other grafts, not elsewhere classified |  |
|  | 90201-03 | Coronary artery bypass, using >= 4 other grafts, not elsewhere classified |  |
|  | 38456-19 | Other intrathoracic procedures on arteries of heart without cardiopulmonary bypass |  |
| Surgeries with cardiopulmonary bypass | 38653-01 | Other intrathoracic procedures on atrium with cardiopulmonary bypass |  |
|  | 38653-02 | Other intrathoracic procedures on ventricle of heart with cardiopulmonary bypass |  |
|  | 38653-03 | Other intrathoracic procedures on septum with cardiopulmonary bypass |  |
|  | 38653-04 | Other intrathoracic procedures on aortic valve with cardiopulmonary bypass |  |
|  | 38653-05 | Other intrathoracic procedures on mitral valve with cardiopulmonary bypass |  |
|  | 38653-06 | Other intrathoracic procedures on tricuspid valve with cardiopulmonary bypass |  |
|  | 38653-07 | Other intrathoracic procedures on pulmonary valve with cardiopulmonary bypass |  |
|  | 38653-08 | Other intrathoracic procedures on arteries of heart with cardiopulmonary bypass |  |
|  | 38600-00 | Cardiopulmonary bypass, central cannulation |  |
|  | 38603-00 | Cardiopulmonary bypass, peripheral cannulation |  |
|  | 38627-01 | Adjustment of cannula for cardiopulmonary bypass |  |
|  | 38653-00 | Other intrathoracic procedures on heart with cardiopulmonary bypass |  |

Footnote: In-hospital complications were identified by procedure codes and secondary diagnoses of the index hospitalisation. Post-discharge complications were identified by the procedure codes and the primary diagnosis of hospital readmissions.

ACHI = Australian Classification of Health Interventions; AMI = acute myocardial infarction; ICD10-AM = International Classification of Diseases, 10^th^ Revision, Australian Modification.

**Table S3: Rates of in-hospital and post-discharge complications stratified by hospital’s type**

| **Procedural complications** | **In-hospital complications** | | | **Post-discharge complications** | | |
| --- | --- | --- | --- | --- | --- | --- |
|  | **Public hospitals**  **n (%)** | **Private hospitals**  **n (%)** | **P value** | **Public hospitals**  **n (%)** | **Private hospitals**  **n (%)** | **P value** |
| Death | 2 (0.04) | 2 (0.01) | *0.205* | 2 (0.04) | 8 (0.05) | *1.000* |
| Cardiopulmonary failure and shock | 19 (0.41) | 17 (0.10) | *<0.001* | 0 (0.00) | 3 (0.02) | *1.000* |
| Stroke/TIA | 8 (0.17) | 16 (0.09) | *0.159* | 2 (0.04) | 23 (0.14) | *0.100* |
| Pericardial effusion | 42 (0.90) | 78 (0.46) | *<0.001* | 9 (0.19) | 12 (0.07) | *0.029* |
| Haemothorax/pneumothorax | 6 (0.13) | 10 (0.06) | *0.130* | 0 (0.00) | 14 (0.08) | *0.051* |
| Any bleeding | 152 (3.26) | 408 (2.40) | *0.001* | 37 (0.79) | 97 (0.57) | *0.086* |
| Vascular injury or intervention | 8 (0.17) | 18 (0.11) | *0.251* | 7 (0.15) | 15 (0.09) | *0.295* |
| Postprocedural infections | 15 (0.32) | 27 (0.16) | *0.025* | 12 (0.26) | 38 (0.22) | *0.670* |
| Pericarditis | 20 (0.43) | 30 (0.18) | *0.001* | 5 (0.11) | 10 (0.06) | *0.340* |
| Procedure-related AMI | 1 (0.02) | 8 (0.05) | *0.694* | 4 (0.09) | 9 (0.05) | *0.496* |
| Venous thromboembolism | 2 (0.04) | 5 (0.03) | *0.648* | 1 (0.02) | 8 (0.05) | *0.694* |
| Acute kidney injury | 30 (0.64) | 20 (0.12) | *<0.001* | 0 (0.00) | 4 (0.02) | *0.584* |
| Complications requiring cardiac surgery | 8 (0.17) | 6 (0.14) | *0.004* | 3 (0.06) | 6 (0.04) | *0.416* |
| Complete atrioventricular block | 11 (0.24) | 37 (0.22) | *0.815* | 0 (0.00) | 3 (0.02) | *1.000* |

Footnote: TIA = transient ischaemic attack, AMI = acute myocardial infarction.

**Table S4: Variables included in the logistic regression model to evaluate the association between ablation at a public hospital and risk of complications (treatment at a private hospital as the reference group).**

| **Variables** | **OR** | **SE** | **P value** | **95% CI** |
| --- | --- | --- | --- | --- |
| Ablation at a public hospital | 1.77 | 0.13 | <0.001 | 1.54 – 2.04 |
| Female | 1.22 | 0.08 | 0.002 | 1.08 – 1.39 |
| Age | 1.02 | 0.00 | <0.001 | 1.01 – 1.02 |
| History of catheter ablation in the preceding year | 0.78 | 0.08 | 0.012 | 0.64 – 0.95 |
| Ablation of both atria | 1.60 | 0.10 | <0.001 | 1.41 – 1.82 |
| Hypertension | 1.40 | 0.12 | <0.001 | 1.18 – 1.66 |
| Haematological disorders | 2.33 | 0.25 | <0.001 | 1.88 – 2.89 |
| History of pneumonia | 2.32 | 0.34 | <0.001 | 1.74 – 3.10 |
| Musculoskeletal and connective tissue disorders | 1.31 | 0.13 | 0.007 | 1.07 – 1.59 |

Footnote: OR = adjusted odd ration, SE=standardised error, CI = confidence intervals.

**Table S5: Association between treatment at a public hospital and the risk of experiencing in-hospital and post-discharge complications with logistic regression (treatment at a private hospital as the reference group).**

| **Complications** | **In-hospital complications** | | **Post-discharge complications** | |
| --- | --- | --- | --- | --- |
|  | **OR and 95% CI** | **P value** | **OR and 95% CI** | **P value** |
| Death | 1.81 (0.23 – 14.25) | 0.574 | 0.72 (0.15 – 3.53) | 0.683 |
| Cardiorespiratory failure | 3.94 (1.97 – 7.89) | <0.001 | NA | NA |
| Stroke/TIA | 2.02 (0.82 – 4.97) | 0.124 | 0.35 (0.08 – 1.54) | 0.165 |
| Pericardial effusion | 2.09 (1.40 – 3.11) | <0.001 | 3.06 (1.12 – 7.67) | 0.017 |
| *Pericardiocentesis* | 1.86 (1.08 – 3.20) | 0.026 | 2.36 (0.78 – 7.09) | 0.127 |
| Hemothorax/pneumothorax | 2.55 (0.88 – 7.38) | 0.084 | NA | NA |
| Bleeding | 1.56 (1.28 – 1.91) | <0.001 | 1.48 (1.00 – 2.20) | 0.052 |
| *Postprocedural hemorrhage or hematoma* | 1.59 (1.28 – 1.98) | <0.001 | 1.83 (1.01 – 3.30) | 0.046 |
| *Bleeding from other sites* | 1.35 (0.83 – 2.20) | 0.226 | 1.04 (0.44 – 2.49) | 0.924 |
| *Bleeding requiring blood transfusion* | 1.44 (0.75 – 2.78) | 0.278 | 1.14 (0.59 – 2.19) | 0.692 |
| Vascular injury | 1.59 (0.67 – 3.75) | 0.291 | 1.90 (0.74 – 4.87) | 0.181 |
| Postprocedural infections | 1.85 (0.91 – 3.76) | 0.088 | 1.25 (0.64 – 2.45) | 0.518 |
| Pericarditis | 2.38 (1.30 – 4.36) | 0.005 | 2.55 (0.83 – 7.85) | 0.104 |
| Acute myocardial infarction | 0.41 (0.05 – 3.36) | 0.407 | 1.91 (0.56 – 6.49) | 0.302 |
| Venous thromboembolism | 1.77 (0.31 – 9.98) | 0.517 | 0.52 (0.06 – 4.28) | 0.544 |
| Acute kidney injury | 6.20 (3.42 – 11.26) | <0.001 | NA | NA |
| Complications requiring cardiac surgery | 6.98 (2.29 – 21.29) | 0.001 | 3.14 (0.75 – 13.17) | 0.117 |
| Complete AV block | 1.05 (0.53 – 2.08) | 0.894 | NA | NA |

Footnote: TIA=Transient ischaemic attack, AV=atrioventricular, NA=Not applicable (event rate was 0 in one of two groups).
